# The BET protein FSH functionally interacts with ASH1 to orchestrate global gene activity in Drosophila

**DOI:** 10.1186/gb-2013-14-2-r18

**Published:** 2013-02-25

**Authors:** Tobias Kockmann, Moritz Gerstung, Tommy Schlumpf, Zhu Xhinzhou, Daniel Hess, Niko Beerenwinkel, Christian Beisel, Renato Paro

**Affiliations:** 1 ETH Zurich, Department of Biosystems Science and Engineering (D-BSSE), Mattenstrasse 26, 4058 Basel, Switzerland; 2 SIB Swiss Institute of Bioinformatics, Lausanne, Switzerland; 3 Wellcome Trust Sanger Institute, Wellcome Trust Genome Campus, Hinxton, Cambridgeshire, CB10 1SA UK; 4 Friedrich Miescher Institute for Biomedical Research, Maulbeerstrasse 66, 4058 Basel, Switzerland; 5 Faculty of Science, Klingelbergstrasse 50, 4056 Basel, Switzerland

**Keywords:** *Drosophila melanogaster*, Trithorax group (TrxG), Absent, small, or homeotic discs 1 protein (ASH1), [Swiss-Prot:Q9VW15], Female sterile (1) homeotic protein (FSH), [Swiss-Prot:P13709], transcriptional regulation, epigenetic gene control

## Abstract

**Background:**

The question of how cells re-establish gene expression states after cell division is still poorly understood. Genetic and molecular analyses have indicated that Trithorax group (TrxG) proteins are critical for the long-term maintenance of active gene expression states in many organisms. A generally accepted model suggests that TrxG proteins contribute to maintenance of transcription by protecting genes from inappropriate Polycomb group (PcG)-mediated silencing, instead of directly promoting transcription.

**Results and discussion:**

Here we report a physical and functional interaction in *Drosophila *between two members of the TrxG, the histone methyltransferase ASH1 and the bromodomain and extraterminal family protein FSH. We investigated this interface at the genome level, uncovering a widespread co-localization of both proteins at promoters and PcG-bound intergenic elements. Our integrative analysis of chromatin maps and gene expression profiles revealed that the observed ASH1-FSH binding pattern at promoters is a hallmark of active genes. Inhibition of FSH-binding to chromatin resulted in global down-regulation of transcription. In addition, we found that genes displaying marks of robust PcG-mediated repression also have ASH1 and FSH bound to their promoters.

**Conclusions:**

Our data strongly favor a global coactivator function of ASH1 and FSH during transcription, as opposed to the notion that TrxG proteins impede inappropriate PcG-mediated silencing, but are dispensable elsewhere. Instead, our results suggest that PcG repression needs to overcome the transcription-promoting function of ASH1 and FSH in order to silence genes.

## Background

Gene expression programs specify diverse cellular identities during metazoan development, ultimately allowing cells to form tissues or organs. The ability to maintain expression states is critical, since inappropriate loss or gain of gene activity may lead to developmental anomalies, tissue dysfunction, or uncontrolled cell growth such as cancer. The Trithorax group (TrxG) proteins and the Polycomb group (PcG) proteins, originally identified in *Drosophila melanogaster *but present in all higher eukaryotes, cooperate to sustain gene expression states by establishing and organizing information contained in the chromatin template. This epigenetic information layer assures the appropriate usage of the genetic blueprint, according to the developmental history of any given cell. TrxG proteins are required in order to maintain active states. This was discovered because of their essential *trans*-activating function for homeotic gene expression in flies [1]. Despite their common implication in gene activation, the TrxG encompasses a variety of different biochemical functions, ranging from chromatin remodeling and histone modification to mediator complex subunits and transcription factors [2]. Genetically, the TrxG acts antagonistic to the PcG of genes, which is essential for the maintenance of repressed expression states.

The histone methyltransferases (HMTs) absent, small, or homeotic discs 1 (*ash1*) and *trithorax (trx) *represent histone modifying activities within the TrxG. Both HMTs have attracted particular attention, because they were considered to specifically counteract PcG silencing, rather than being coactivators of transcription [3]. Studies of ASH1 enzymatic activity supporting this view show that the histone modifications catalyzed by ASH1 disfavor Polycomb Repressive Complex 1 (PRC1) binding *in-vitro*, whereas association of SWI/SNF chromatin remodeling complexes incorporating TrxG subunits (BRAHMA, MOIRA) is preferred [4]. However, the *in-vivo *relevance of this observation remains unclear, since the targeting of PRC1 seems to primarily depend on transcription factors and non-coding RNAs (ncRNAs) [5]. In addition, the assumed enzymatic specificity of ASH1 has lately been challenged by independent reports [6-8].

Alternative explanations on how ASH1 prevents PcG-mediated silencing propose that ASH1 blocks PRC-activity downstream to chromatin recruitment. A comparison of the HOX gene *Ultrabithorax (Ubx*) in active and repressed conditions supports this notion: PRC1 and PRC2 binding patterns at the *Ubx *locus do not change between *Ubx *ON or OFF states, whereas ASH1 is only located downstream of the active *Ubx *promoter in embryonic discs [9]. In addition, *Ubx *repression in the absence of ASH1 is accompanied by the formation of an ectopic H3K27me3 domain, a mark associated with mature PcG-mediated repression. Taken together, these data suggest that ASH1 actively prevents PcG silencing, however not by simply impairing PRC1/2 recruitment to chromatin.

The functional relationship between PcG proteins and ASH1 has been addressed on a genome-wide scale by two recent ChIP-chip studies in *Drosophila *tissue culture cells. Both investigations support the proposed anti-repressor function of ASH1, either by showing that fully activated PcG target genes miss typical marks of PcG-mediated repression and instead become embedded in broad ASH1 domains [10], or by reporting that genes in 'balanced' states, characterized by simultaneous binding of PcG and TrxG proteins, are transcriptionally active [10,11]. In both studies a clear indication for a more general involvement of ASH1 in transcription is missing. In accordance to this, analysis of the wing imaginal disc transcriptome of *ash1 *mutant flies only identified few deregulated genes, again supporting the idea that ASH1 is only needed at a subset of developmental regulators, which need to be protected from ectopic PcG repression [12].

Curiously, studies analyzing *Ubx *pattern formation in embryonic disc stages suggest ASH1 to act as a general transcription-promoting factor in analogy to what has been shown for TRX. Briefly, ncRNAs, originating from the *bxd *region upstream of the *Ubx *promoter, inhibit *Ubx *expression in *cis *presumably by transcriptional interference [13]. ASH1 localization downstream of the *bxd *ncRNA promoters correlates with the onset of ncRNA expression [14]. This behavior is reminiscent of TRX, which was already shown to promote *bxd *ncRNA expression, thereby silencing *Ubx *indirectly [15]. In the reverse *Ubx *ON situation, ASH1 was found to be enriched in the coding region of *Ubx *and a related GFP reporter gene, but absent from *bxd ncRNAs*. Hence, the correlation between ASH1 binding and transcriptional activity at functionally diverse genes (ncRNAs, homeotic selector, and reporter) is clearly indicative of a more general involvement in transcription.

Another TrxG member, explicitly implicated in *Ubx *regulation, is female sterile (1) homeotic (*fs(1)h*) [16,17]. Alleles of *ash1 *and *fs(1)h *show strong genetic interactions with respect to *Ubx *activity. These interactions have contributed to the proposal of a 'trithorax gene set' by Shearn, following the earlier idea of a repressive gene cohort around Polycomb [18]. However, attempts to link ASH1 and FSH on a biochemical level have not been successful to date [17]. The *fs(1)h *gene products belong to the BET family of proteins, named after their characteristic arrangement of a tandem bromodomain and the extra-terminal domain. Mammalian BET proteins have been shown to serve as chromatin adapter proteins by binding to acetylated histone tails and to facilitate gene expression [19]. Lately, inhibition of BET function has been identified as effective strategy to treat poor-prognosis leukemia, multiple myeloma, and squamous carcinomas [20-23]. How BET proteins contribute to normal development and tissue homeostasis is still poorly understood. Loss of function phenotypes in model organisms having a single BET gene, such as *C. elegans *BET-1 mutants and *Drosophila fs(1)h *mutants, demonstrate that BET function is crucial for the establishment and maintenance of cell fates [16,24,25].

By purifying ASH1 from a stable *Drosophila *cell line, we discovered a biochemical interaction between ASH1 and FSH, as previously suggested from genetic studies in fly mutants. In addition, we were able to show that both proteins extensively co-localize on chromatin, especially at sites bound by PcG complexes and gene promoters. The observed co-localization at promoters is a hallmark of active genes, but independent of gene type and function. These findings are at odds with the prevalent model that TrxG proteins function primarily as PcG-specific anti-repressors, but are dispensable in the absence of PcG-mediated repression. Our additional finding, that a set of PcG-repressed genes displays ASH1, FSH, and TRX-C signals comparable to their active counterparts, further questions the anti-repressor hypothesis. In agreement with a general transcription promoting function, we could show that BET inhibition in *Drosophila *tissue culture cells leads to an immediate and widespread gene repression, as well as delayed gene activation in reaction to environmental stimuli.

## Results

### ASH1 interacts biochemically with FSH

**Figure 1:**
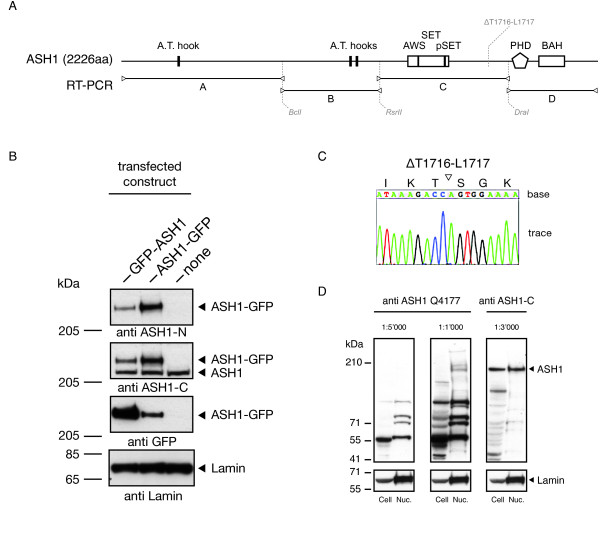
**Cloning and functional verification of a full-length ASH1 cDNA**. (**A**) Map of *Drosophila *ASH1 protein indicating known domains [SwissProt:Q9VW15]. Subcloned RT-PCR amplicons used for cDNA assembly are shown below. (**B**) ASH1-specific antibodies, raised against epitopes residing in the N- and C-terminal portion of the protein, detect ASH1-GFP fusion products obtained by transfecting *Drosophila *cells with expression constructs stated on top. (**C**) Sanger sequencing trace indicating missing amino acids T1716-L1717 in cloned ASH1 cDNA. (**D**) Comparision of target and cross reactivity between the ASH1-specific antibodies ASH1-C and Q4177. Immunoblots of S2-DRSC derived whole cell (Cell) and nuclear lysate (Nuc.) were prepared using the antibody dilutions given on top.

In order to screen for ASH1-interacting proteins, we purified tandem-tagged ASH1 from a stable *Drosophila *cell line and identified copurified proteins by tandem mass spectrometry (MS/MS). Since no full-length cDNA was available to generate bait expression constructs, we cloned the *ash1 *coding sequence from S2 cells by assembling four subcloned RT-PCR products (A-D) tilling the ASH1 open reading frame (ORF) from start to stop codon (Figure 1A). We verified the resulting cDNA by transfecting cells with ASH1-GFP fusion constructs. Immunoblotting of cell lysates gave clearly detectable signals at the expected molecular weight (MW) using antibodies (kind gift from F. Sauer, UC Riverside) recognizing epitopes in the N- and C-terminal part of ASH1 (Figure 1B). Sanger sequencing showed that our cDNA contains a micro deletion of the amino acids (aa) T1716-L1717 with respect to [Swiss-Prot:Q9VW15] (Figure 1C). In spite of the two missing amino acids it still encodes a fully functional protein, as demonstrated by rescuing the development of *ash1 *null mutant flies (Steffen PA, Fonseca JP, Gänger C, Dworschak E, Kockmann T, Beisel C, Ringrose L: Quantitative in vivo analysis of chromatin binding of Polycomb and Trithorax group proteins reveals retention of ASH1 on mitotic chromatin. Nucleic Acids Research 2013, 41:5235-5010.1093/nar/gkt217Available: http://eutils.ncbi.nlm.nih.gov/entrez/eutils/elink.fcgi?dbfrom=pubmed&id = 23580551&retmode=ref&cmd=prlinks.). An ASH1 ChIP-chip profile has already been published by modENCODE [11]. As a means of comparison we tested the immunoreactivity of the antibody (Q4177) used in their study towards recognizing ASH1, which according to modENCODE targets aa 1747-1846. To our surprise, we could not detect signals corresponding to ASH1 by probing whole cell und nuclear extracts (Figure 1D). Conversely, control immunoblots of the same material faithfully detected ASH1 using the ASH1-C antibody.

**Figure 2:**
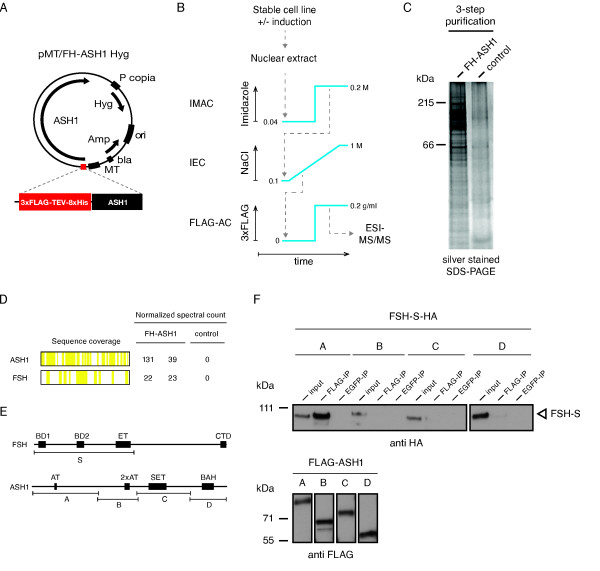
**The TrxG protein FSH interacts biochemically with ASH1**. (**A**) Map of inducible, tandem-tagged ASH1 expression construct used to establish stable cell line. Amp: ampicillin resistance, bla: beta-lactamase promoter; FH: 3xFLAG-8xHIS tandem affinity tag; Hyg: hygromycin resistance; MT: metallothionein promoter;, ori: origin of replication; P copia: copia promoter. (**B**) Outline of ASH1 purification scheme. ESI-MS/MS: electrospray ionization tandem mass spectrometry; FLAG-AC: FLAG affinity chromatography; IEC: ion exchange chromatography; IMAC: immobilized metal affinity chromatography. (**C**) FH-ASH1 dependent enrichment of proteins by purification scheme. Purified FH-ASH1 sample was size separated and silver stained together with control sample. (**D**) Summary of mass spectrometry results; only the top two enriched proteins are shown (for complete results see Additional file 1). (**E**) Maps of protein fragments used for coimmunoprecipitation experiments. (**F**) Coimmunoprecipitation of FSH-S through ASH-A fragment. S2-DRSC cells were cotransfected with the indicated constructs, and whole cell lysates and precipitated proteins were immunoblotted using the indicated antibodies.

Based on our novel ASH1 cDNA we created a double-affinity tagged expression construct under the control of the inducible metallothionein promoter (Figure 2A). Employing this vector construct we established a polyclonal S2-DRSC cell line, exhibiting inducible expression of 3xFLAG-8xHIS-tagged ASH1 (FH-ASH1). Following overnight induction of FH-ASH1, a three-step purification as outlined in Figure 2B was performed. In the first step, we captured tagged ASH1 from nuclear extract by immobilized metal affinity chromatography (IMAC). Captured FH-ASH1 was afterwards subjected to ion exchange chromatography (IEX) and finally to FLAG-affinity chromatography (FLAG-AC) for polishing. In order to test if our purification scheme enriched proteins in a bait dependent manner, we simultaneously size-separated samples originating from induced and non-induced cells. Silver staining visualized many protein bands exclusively present in the experimental sample, indicating a bait protein dependent enrichment (Figure 2C).

MS/MS analysis revealed that FH-ASH1 was strongly enriched in the experimental samples, but absent from non-induced control samples (Figure 2D and Additional file 1). The second most enriched protein was identified to be female sterile (1) homeotic [Swiss-Prot:P13709], encoded by the *bona fide *TrxG gene *fs(1)h*. Alternative splicing of the primary *fs(1)h *transcript results in two polypeptides: a short isoform denoted FSH-S, and a long isoform denoted FSH-L. The second is a C-terminal extension of its shorter relative. In order to approximately map the ASH1-FSH interaction, we co-expressed the ASH1 fragments A-D in combination with FSH-S in S2-DRSC cells. Immunoprecipitation of ASH1 A proved to be sufficient to pull-down FSH-S (Figure 2F), suggesting that the ASH1-interaction motive resides in the common N-terminal portion of the two splice products. These results agree with the FSH peptide coverage from our MS/MS analysis, since we also found peptides belonging to the long isoform.

**Figure 3:**
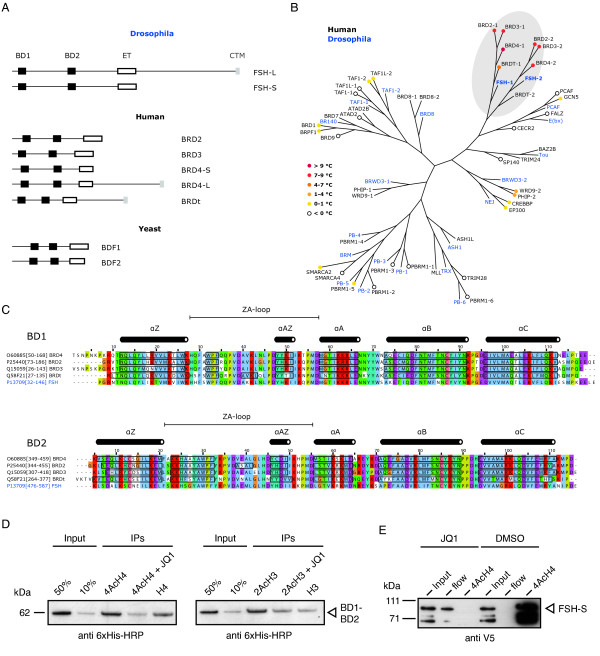
**The BET family inhibitor JQ1 specifically targets FSH in *Drosophila***. (**A**) Conserved protein domain arrangement between BET family proteins. The *Drosophila *BET family is exclusively represented by the long and short isoform of FSH. BD1: Bromodomain 1; BD2: Bromodomain 2; CTM: C-terminal motive; ET: Extraterminal domain. (**B**) Assesment of BET inhibitor selectivity using a phylogenetic analysis of *Drosophila *(blue) and human (black) bromodomains. BRD and FSH bromodomains form the BET clade highligted in gray. Notecolors of human proteins indicate averaged temperature shifts upon binding of 10 µM JQ1 measured by differential scanning fluorimerty [20]. (**C**) Protein sequence alignment of human and *Drosophila *BET bromodomains. Boxed residues reside in α helices as determined by structural analysis [26]. The high degree of sequence conservation suggests that FSH bromodomains shape into the typical left-handed bundle of four α helices (α_Z_, α_A_, α_B_, α_C_) connected by loop regions. (**D**) Histone peptide pull-down assays using a purified, 6xHis-tagged BD1-BD2 fragment from FSH. 10 µM JQ1 effectively eliminates the interaction between acetlyted H4 (K5, K8, K12, K16) or H3 (K9, K14) peptides and the di-bromodomain fragment. (**E**) Similar histone peptide pull-down assay using nuclear extract from HEK293 cells transfected with V5-tagged FSH-S.

The unique domain arrangement identifies FSH as members of the bromodomain and extraterminal (BET) family. BET proteins are conserved from humans to lower eukaryotes and have been shown to regulate gene expression by means of chromatin (Figure 3A). Their N-terminal region contains a tandem bromodomain which attaches BET proteins to acetylated histones. The tandem bromodomain is followed by the extraterminal domain of unknown function, which is likewise present in all BET proteins. The human BET family is comprised of BRD2, 3, 4, and BRDt. *Drosophila *FSH-L is closest to BRD4 and BRDt, since all three proteins possess the C-terminal tail extension. This tail is characterized by an unusual amino acid composition, for instance including poly-glutamine runs, and a C-terminal motive (CTM) at the very end of the protein. FSH-S matches BRD2, 3, and BRD4-S with respect to the missing tail region.

Large-scale analysis has shown that human bromodomains cluster into eight families based on structure/sequence similarity [26]. Family II is populated by the BET-type bromodomains of BRD2, 3, 4, and BRDt. The recent development of the highly potent, small molecule inhibitor JQ1 allows selective inhibition of BET bromodomains [20,27]. JQ1 specificity is explained by the excellent shape complementarity with the acetyl-lysine binding cavity of BET-type bromodomains. We therefore asked, if a similar selectivity can be expected in the context of the *Drosophila *model system. Analogous to the situation in humans, the fruit fly proteome contains several bromodomain proteins, which have been implicated in transcriptional control. We approached this question by a phylogenetic analysis including human bromodomains of known drug ability and their *Drosophila *homologs. We found that FSH bromodomains are highly conserved with respect to BRD proteins (approximately 80% identity, >95% similarity), but well separated from bromodomains of other families (Figure 3B). The superimposed human JQ1 selectivity strongly argues in favor of a discriminating drug ability of FSH. Structural alignments predict that FSH bromodomains fold into the canonical left-handed bundle of four α helices (α_Z_-α_A_-α_B_-α_C_) creating a hydrophobic acetyl lysine binding pocket (Figure 3C). All residues creating side-chain contacts to JQ1 have been found conserved.

In order to assess whether JQ1 binding is competitive with acetyl-lysine recognition, we performed histone peptide pull-down assays. Binding of tetra-acetylated histone H4 tail peptide to a purified BD1-BD2 fragment was reduced to background levels in the presence of 10 µM JQ1 (Figure 3D). We made similar observations using a dual acetylated H3 tail peptide. Binding of none-purified FSH-S, obtained by transfecting HEK293 cells, was likewise suppressed by JQ1 (Figure 3D). We therefore conclude that JQ1 can be used to selectively target BET function in *Drosophila*.

### ASH1 and FSH co-localize on chromatin

**Figure 4:**
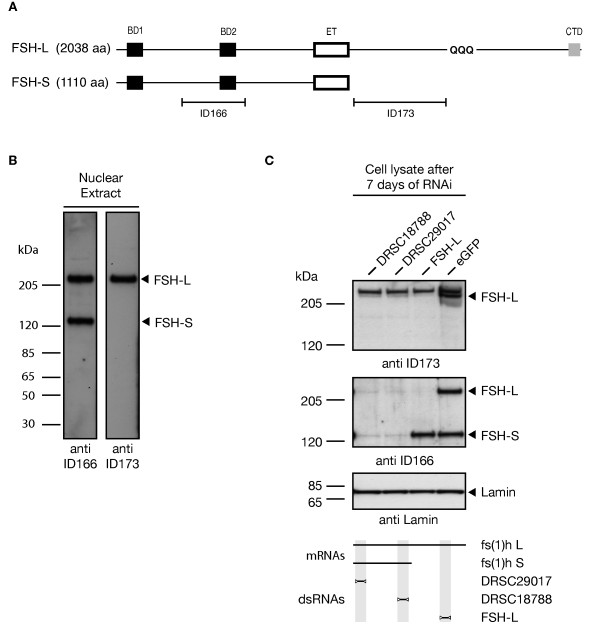
**FSH antibody preparation and validation of specificity**. (**A**) Map of FSH protein isoforms indicating location of antigens ID166 and ID173 used for rabbit immunization. BD1: bromodomain 1; BD2: bromodomain 2; CTD: C-terminal domain; ET: extraterminal domain. (**B**) Nuclear extract from S2-DRSC cells was immunoblotted using FSH-specific antibodies; arrowheads indicate detected FSH isoforms. (**C**) S2-DRSC cells were depleted from FSH isoforms using RNAi; immunoblotting of corresponding cell lysates using FSH-specific antibodies demonstrates the expected specificity.

Binding sites of ASH1 to chromosomes have been mapped on a genome-scale in different *Drosophila *cell lines using ChIP-chip methodology, while maps of FSH chromatin occupancy are not available to date. In order to clarify whether ASH1 and FSH also interact on chromatin, we performed ChIP-seq experiments in S2-DRSC cells. In addition, we contrasted their binding profiles with genome-scale maps for polycomb (PC), polyhomeotic (PH), posterior sex combs (PSC), and the C-terminal TaspaseI cleavage product of Trithorax (TRX-C), generated previously by us [28]. We enriched for ASH1-bound chromatin by using the antibodies ASH1-N and ASH1-C. FSH-bound chromatin was precipitated by antibody preparations from immune sera kindly provided by Igor B. Dawid (NICHD). Antibody ID166, raised against an immunogen in the common N-terminal part of FSH, is reactive against both FSH isoforms (Figure 4A, B). ID173 can be used to exclusively detect FSH-L. We verified antibody specificity by performing *fs(1)h *knockdown experiments in S2-DRSC cells (Figure 4C).

**Figure 5:**
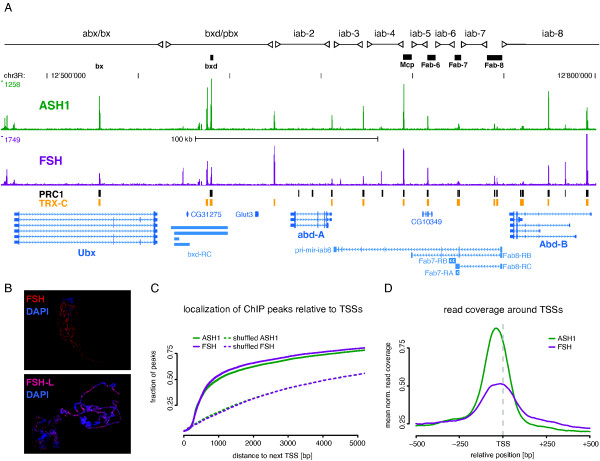
**ASH1 and FSH extensively co-localize on chromatin**. Genome-wide maps of ASH1 and FSH chromatin occupancy were generated using ChIP-seq. (**A**) Modified UCSC genome browser view of the bithorax gene cluster (BX-C) in S2-DRSC cells shows ASH1, FSH co-localization at TRX-C, PRC1-bound sites. The position of regulatory iab regions (capped bars) and cloned PREs (solid boxes) are indicated above track area. ASH1 and FSH tracks show coverage profiles calculated from aligned ChIP-seq reads; solid boxes of PRC1 track (black) depict regions significantly co-enriched for the PRC1 core subunits PC, PH, and PSC; the TRX-C track (yellow) displays intervals enriched for the C-terminal fragment of TRX. FlyBase gene models and non-coding RNAs are shown below track area. (**B**) FSH-specific immunostainings of *Drosophila *salivary gland chromosomes. (**C**) ASH1 and FSH peaks are closer to TSS then expected by chance. Graph shows empirical cumulative density functions (ECDFs) for the distance between identified ASH1, FSH peaks and the closest TSS. For comparison, the ECDFs calculated form shuffled peak intervals are plotted as dashed lines. (**D**) Accumulation of ASH1, FSH ChIP-seq signal around TSS. Graph displays normalized read coverage for ASH1, FSH averaged over 1-kb windows centered at known TSS.

Since ASH1 and FSH are known *trans*-activators of homeotic gene expression, with central importance for *Ubx *activation and maintenance [16,29], we first focused on the bithorax complex (BX-C). We found that ASH1 and FSH co-localize at several discrete sites throughout the BX-C (Figure 5A). The great majority of bound sites coincide with known boundary elements, which divide the gene cluster into regulatory domains [30]. But also known *cis*-regulatory elements like the *Ubx *enhancers *bx *and *bxd *show strong ASH1, FSH co-enrichments. Despite their presence throughout the BX-C, all genes of the homeotic cluster are silent in S2-DRSC cells. Considering their *trans *activating function, this finding is contrary to expectation.

Our comparison with the additionally mapped PcG/TrxG factors revealed that ASH1, FSH co-localization throughout the BX-C is highly coordinated with PRC1 and TRX-C binding to chromatin (Figure 5A). We defined PRC1-bound chromatin as genomic intervals co-enriched for the PRC1 core subunits PC, PH, and PSC (see Material and Methods for details). Again, this finding is rather surprising since TrxG and PcG proteins implement antagonistic gene regulatory functions. We decided to further investigate these phenomena on a genome scale and found that the observed co-localization is not restricted to the homeotic clusters. In total, we identified about 10,700 regions enriched for ASH1 and about 8,800 occupied by FSH. By using the combined chromatin maps for PC, PH, and PSC we identified 800 sites bound by PRC1 with high confidence. More than 80% of these sites are shared with ASH1 and the same is true for FSH (Table 1). In agreement with our ChIP-seq data, immunostaining of polytene chromosomes visualized hundreds of FSH-bound regions (Figure 5B).

**Table 1:** Overlap between TrxG/PcG ChIP-seq peaks in S2-DRSC cells.

		Fraction (%) of query peaks overlapping^a ^reference peaks			
		
Query	Peaks (*n*)	ASH1	FSH	PRC1	TRX-C
ASH1	10,748	-	82	6	21
FSH	8,824	92	-	7	25
PRC1	800	87	82	-	83
TRX-C	2,608	94	92	24	-

Table lists pair-wise, directional overlap between ASH1, FSH, PRC1, and TRX-C ChIP-seq peaks identified using MACS (see Materials and methods for details).

^a^Peak intervals enriched for the query protein overlap by *≥*1 bp with at least one interval occupied by the reference protein.

We previously showed that gene promoters are major recruitment sites for TrxG/PcG proteins [28]. We therefore examined the spatial relationship between transcription start sites (TSS) and ASH1, FSH bound genomic regions, by measuring the distance between ASH1, FSH peaks and the closest annotated TSS (see Materials and methods for details). As shown in Figure 5C, a majority of peaks are closer to TSS then expected by chance, in fact >50% are within 1 kb of distance (random peak distribution was modeled by shuffling peaks along chromosomes, *P *value <2.2 × 10^-16^). In order to further fine-map ASH1, FSH binding relative to TSSs, we calculated binding profiles across non-overlapping 1 kb windows centered at TSSs. For both proteins we obtained unimodal coverage distributions with a maximum very close to the aligned TSS positions (Figure 5D). These localization profiles demonstrate that ASH1 and FSH prefer binding nearby TSSs.

In summary, ASH1 and FSH binding to chromatin is strongly correlated and seems to happen at many sites in dividing cells, as well as post mitotic tissue. Our high-resolution analysis suggests that these sites coincide with the starting point of transcription. But also intergenic elements bound by the PRC1 complex attract ASH1 and FSH.

### ASH1 and FSH co-localization is a hallmark of active genes

**Figure 6:**
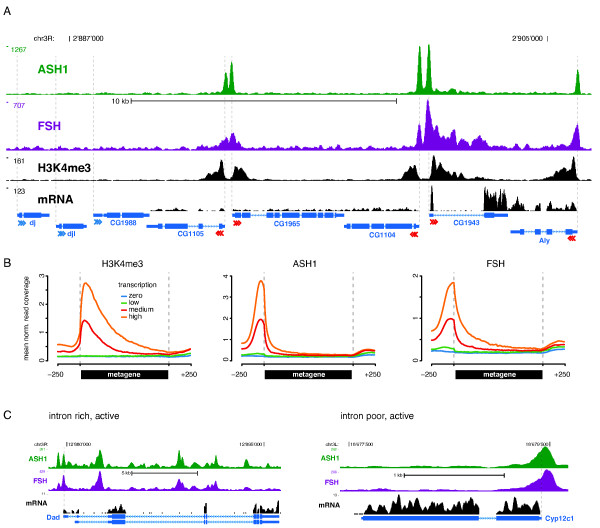
**ASH1, FSH co-localization correlates with gene expression**. (**A**) Modified UCSC genome browser view of a representative genomic region containing silent (blue arrowheads) and active genes (red arrowheads). ASH1 and FSH tracks show data as described before; H3K4me3 track displays read coverage profile for H3K4me3 ChIP-seq experiment; mRNA track contains coverage profile obtained by short-read sequencing of mRNA (RNA-seq). FlyBase gene models are depicted below track area; arrowheads indicate direction of transcription; vertical dashed lines mark TSS positions. (**B**) Graphs show normalized ChIP-seq read density profiles across genes grouped according to their expression level (zero, low, medium, and high). (**C**) Comparison of ASH1, FSH ChIP-seq signals across intron-rich and intron-poor, active genes. Tracks display data as described before. Intron-rich, active genes show strong ASH1, FSH signals inside gene bodies in contrast to their intron-poor counterparts. Both gene classes share promoter proximal signals.

The fact that ASH1 and FSH both bind to promoter regions, prompted us to examine the relationship between gene activity and ASH1, FSH binding in more detail. We did this by inspecting genomic regions containing a mix of silent and transcribed genes. A representative example is shown in Figure 6A. In agreement with our previous results, ASH1 and FSH ChIP-seq signals peak closely to TSSs. A striking difference in ASH1, FSH localization was observed when we compared active and silent genes: All active genes - determined by detectable mRNA expression (RNA-seq) and high H3K4me3 levels at the promoter (ChIP-seq) - displayed prominent ASH1 and FSH peaks close to their TSS. Silent genes, by contrast, lacked these characteristic signals.

We further examined the observed correlation between transcription and ASH1, FSH localization to promoters, by comparing metagene profiles of genes grouped according to their expression level. We refer to a metagene profile as the mean read density over gene bodies scaled to a common length. As reference point for the analysis, we included our H3K4me3 ChIP-seq data, because this chromatin modification is known to be highly correlated with gene expression. In analogy to H3K4me3, the total amount of ASH1, FSH ChIP-seq signal correlated with the magnitude of expression (Figure 6B). The maximum ASH1, FSH signal intensity collapsed with the TSS position when inspecting profiles for active gene bins. To rule out the possibility that our analysis was confounded by a sequencing bias at highly expressed loci, we inspected metagene profiles for our input sample using identical binning, but could not detect indications for such systematic errors (data not shown).

Coverage profiles suggested that ASH1 and FSH normally bind close to the TSS of active genes, but are absent from promoter distal parts of the gene body. Deviating from this canonical observation, we found signals within gene bodies at a subset of active genes: predominantly, these genes fall into the category of intron-rich, active genes. Similar observations have been described earlier [11]. However, the same study did not pick-up the correlation between ASH1 binding and transcription at intron poor genes. Representative examples illustrating the difference between intron-rich and intro-poor active genes are shown in Figure 6C. At the active, intron-rich *Dad *gene, ASH1 and FSH signals seem to spread along the gene body. In contrast, the active intron-poor gene *Cyp12c1 *does not show signals distal to the TSS. Here, the ASH1, FSH signal is tightly restricted to the promoter proximal region.

### Combined TrxG/PcG binding patterns predict the regulatory state of genes

Genetic experiment using double-mutants for TrxG and PcG genes, suggested that TrxG proteins such as ASH1 are primarily needed to prevent inappropriate PcG-mediated silencing, instead of having a general function during transcriptional activation [3]. Since our data clearly favor the second possibility, we decided to examine the combinatorial patterns of TrxG/PcG proteins found at chromatin. The fact that all our ChIP-seq experiments have been carried out using identical experimental procedures/materials ensures high comparability across chromatin maps.

In brief, we identified combinatorial binding patterns by fitting a three component sparse Gaussian mixture model (sGMM) to the ChIP-seq enrichments at promoters (see Materials and methods for details). This approach is related to k-means clustering. Our choice to solely focus on the promoter region was based on our previous finding that all of the TrxG/PcG factors mapped by our laboratory preferred binding close to the TSS. In addition, this special constrain allows for an intuitive correlation between ChIP signals and gene expression.

**Figure 7:**
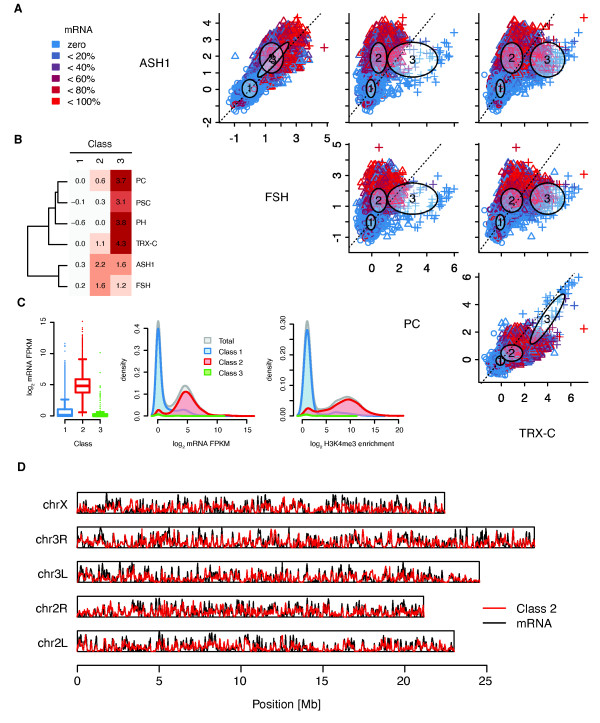
**TrxG/PcG binding profiles are predictive for gene regulatory states**. A sparse Gaussian Mixture Model (sGMM) emitting class assignment (1, 2, and 3) was fitted to ChIP-seq enrichment data for ASH1, FSH, TRX-C, PC, PH, and PSC. Normalized enrichments relative to Input were calculated for the 1-kb window centered at known TSS. (**A**) Pairs plot of the fitted model in selected dimensions; each plotting symbol represents a TSS window (class 1 = circle, class 2 = triangle, class 3 = plus sign); color-coding of plotting symbols indicates corresponding gene expression level (see color key for details). Numbered ellipses denote mean and variance of each class in the plotted dimensions. (**B**) Class 1 genes are characterized by the absence of TrxG/PcG proteins at the promoter. Class 2 and class 3 genes are cobound by ASH1 and FSH, in addition class 3 genes display strong PRC1 and TRX-C enrichments. Heat map plotting class means (columns) over all model dimensions. Row clustering indicates proteins showing correlated changes between classes. (**C**) Class 1 and class2 genes are mostly inactive, while class 2 genes get transcribed. Boxplots display distribution of gene expression within each sGMM class. Graphs show density estimates for gene expression and H3K4me3 modification (1-kb window) within each sGMM class compared to all genes (total). (**D**) Class 2 signal density and mRNA signal density show strong spatial correlation across chromosomes. Plot shows kernel density estimations for class 2 predictions and discretized mRNA signal (log_2 _FPKM cut-off = 2.5) in 1-kb windows tiling chromosomes.

**Figure 8:**
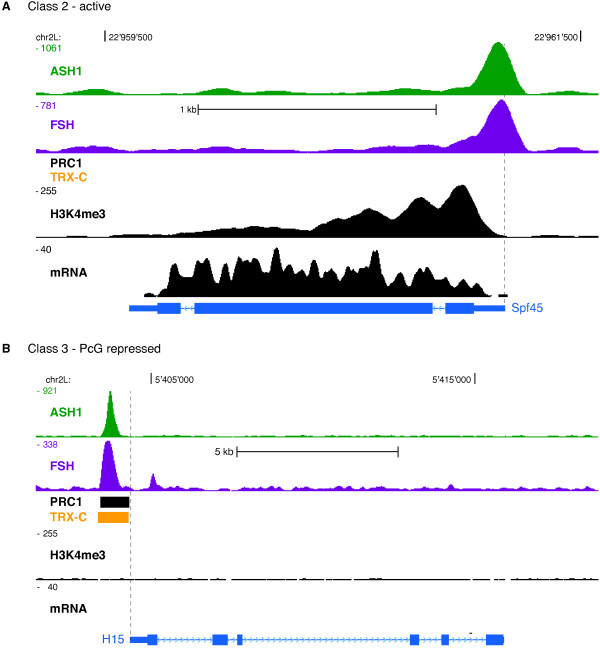
**Comparison of Active and PcG-repressed genes**. (**A**) Genome browser view of the class 2 gene *Spf45*; data are displayed as described before. (**B**) Genome browser view of the class 3 gene *H15*. Both genes are co-bound by ASH1/FSH, whereas PRC1 and TRX-C are selectively enriched at the repressed *H15 *promoter.

The fitted sGMM partitioned genes as follows: class 1 genes do not show enrichments for any of the considered TrxG/PcG proteins at their promoters (Figure 7A, B). Judged by mRNA production the great majority of class 1 genes is not expressed and accordingly lacks the 'active' histone modification H3K4me3 (Figure 7C). In total, approximately 55% of all analyzed genes fall into this class. Therefore, we simply termed this class 'Inactive'. Class 2 genes display strong signals for ASH1 and FSH at their promoters together with intermediate TRX-C enrichments (Figure 7A, B). These genes are clearly transcribed, matching our previous results that ASH1 and FSH presence at promoters correlates with gene expression (Figure 7C). We named this group, containing approximately 43% of all analyzed genes, the 'Active' class. A representative gene belonging to the active cluster is shown in Figure 8A. In agreement with its cluster assignment *Spf45 *displays strong ASH1 and FSH ChIP-seq signals at the transcription start site. The RNA-seq and H3K4me3 ChIP-seq profiles reflect high expression as expected for a splicing factor.

Class 3 genes are characterized by extraordinary high signals in the PC, PH, PSC, and TRX-C dimensions, but their promoters are also occupied by ASH1 and FSH, notably to the same extend as class 2 genes (Figure 7A, B). Approximately 1.5% of all analyzed genes belong to this class. Inspection of their activity showed undetectable expression with very few exceptions (Figure 7C). In combination these findings suggested that class 3 contains genes being actively repressed by the PcG. We therefore termed class 3 'PcG-repressed'. A representative example is given in Figure 8B. The *H15 *TSSs is bound by ASH1, FSH, PRC1, and TRX-C. According to RNA-seq and H3K4me3 signals it is completely repressed. This observation makes sense, since H15 encodes for a transcription factor involved in cardioblast cell fate commitment.

To answer the question how accurately our classification predicts gene expression in a simplified binary manner (ON or OFF), we labeled genes according to their mRNA score as silent or transcribed (log_2 _FPKM cut-off = 2.5) and inspected label frequencies within classes 2 and 3. The cut-off was chosen according to the bimodal distribution of expression values as shown in Figure 7C. For the active class we found that approximately 88% of the genes are expressed according to our cut-off. In case of the PcG-repressed class the inverse is true, meaning approximately 85% of examined genes are silent. Taken together, these frequencies demonstrate that our unsupervised clustering approach has identified patterns that can be used to predict simplified gene expression states with high accuracy. In contrast to former attempts to predict gene expression based on chromatin binding profiles, we did not use any cotranscriptional histone modifications [31,32].

Our results suggest that the class 2 TrxG/PcG signature is a good predictor for active promoters. In order to confirm this, we simply divided the genome into overlapping tiles of 1 kb and examined the correlation between RNA production and class 2 affiliation based on our fitted model (see Materials and methods for details). The genome-wide correlation coefficient of 0.7 indicates that the appearance of class 2 entities and transcription are strongly coupled. To visualize the spatial correlation we co-plotted smoothed density estimates for mRNA production and class 2 predictions along chromosomes (Figure 7D). As suggested by the overall correlation coefficient, both estimates are highly correlated.

### Pharmacologic inhibition of FSH function causes global down-regulation of gene expression

**Figure 9:**
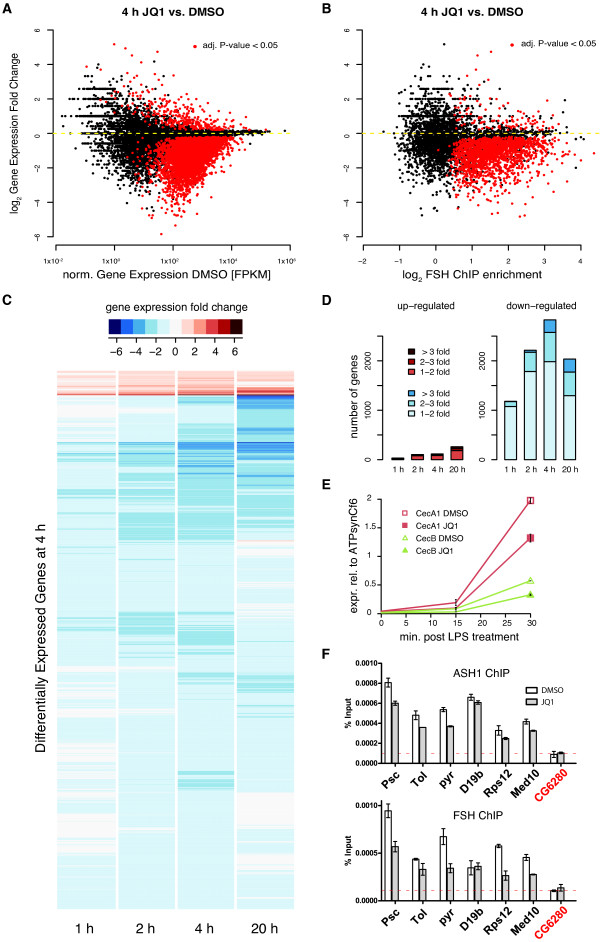
**Small molecule inhibition of FSH induces widespread down-regulation of gene expression**. S2-DRSC cells were treated using the BET inhibitor JQ1 for 1, 2, 4, and 20 h. For each time-point relative gene expression changes were monitored by mRNA-seq. (**A**) MA plot showing gene expression changes after 4 h of JQ1 treatment. Red color highlights genes showing a significant differential expression (adjusted *P *value <0.05). (**B**) Scatterplot of gene expression changes *versus *FSH enrichments at promoters. (**C**) Heat map illustrating expression changes over time for all genes >2 times up- or down-regulated after 4 h of treatment. Colors indicate regularized log_2 _fold change according to color key. (**D**) Bar plots summarizing the number of up- and down-regulated genes for each time point. (**E**) JQ1 pretreatment of S2-DRSC cells dampens LPS-inducible gene expression. *CecA1 *and *CecB *transcript levels were measured using qPCR. (**F**) Removal of FSH from active promoters by JQ1 treatment is accompanied by a loss of ASH1. Bar charts show ChIP signals measured by qPCR. Error bars indicate STDs of two biological replicates. The TSS of CG6280 is neither occupied by ASH1, nor by FSH according to ChIP-seq analysis (represents background signal).

Our chromatin profiling suggests that ASH1 and FSH operate as global transcriptional co-activators irrespective of gene type and function. In order to test this hypothesis, we perturbed FSH binding to chromatin by using the BET inhibitor JQ1 [20]. To test the effect of FSH inhibition on gene expression, we treated exponentially growing S2-DRSC cells with JQ1 for 1, 2, 4, and 20 h and monitored gene expression changes over time by RNA-seq. At 4 h post JQ1 addition approximately 2,500 genes were down-regulated to <50% of their initial level (Figure 9A, C, and D). In sharp contrast, only very few genes reacted to the treatment by elevating transcription. Most genes that were down-regulated at 4 h were already repressed at earlier time points and stayed repressed during the monitored time frame, suggesting a fast and sustainable reduction of gene activity by JQ1 treatment (Figure 9C). Some genes were progressively repressed over time and some reverted to their initial steady-state level at 20 h. The last observation might indicate that these genes find ways to compensate the loss of FSH activity. An alternative explanation could be the propagation of secondary effects in the regulatory network. Plotting gene expression fold-changes *versus *FSH enrichments at promoters visualized that down-regulated genes are bound by FSH in the absence of drug treatment (Figure 9B).

We next asked how the withdrawal of FSH from chromatin affects the localization of ASH1. We addressed this question by treating S2-DRSC cells with JQ1 for 4 h and monitoring the chromatin association of both proteins to a selection of active promoters using ChIP-qPCR. In agreement with the previous result the treatment induced a graduate loss of FSH from active promoters. At most examined sites, FSH is reduced to approximately 50% of the initial level, but there are also insensitive loci, which show no or minor changes upon BET inhibition (Figure 9F). The reduction of FSH occupancy at promoters is accompanied by a limited loss of ASH1 from the same locations. This correlation might suggest that FSH helps to stabilize the interaction of ASH1 with target promoters. Alternatively, altered ASH1 levels at promoters simply reflect reduced transcription.

The reported experiments focused on the relevance of FSH function for steady-state transcription. In addition, we wondered whether FSH is needed for the effective activation of gene expression in response to stimuli. In order to test this, we exposed S2-DRSC cells to lipopolysaccharide (LPS), which is known to trigger the expression of genes coding for antimicrobial peptides (AMPs). Cells pretreated with JQ1 showed a slower accumulation of the AMP transcripts *CecA1 *and *CecB *in response to LPS induction (Figure 9E).

### RNAi-mediated knockdown of FSH induces minor gene expression changes with repespect to pharmacological treatment

**Figure 10:**
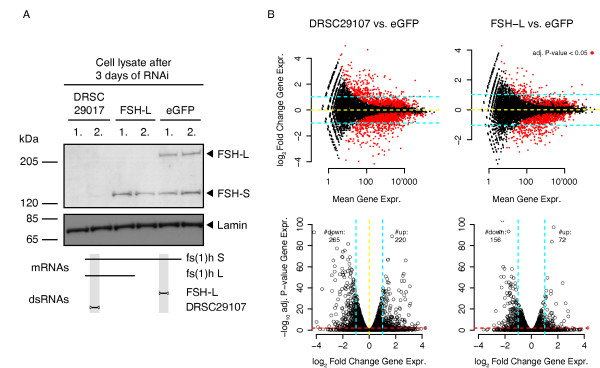
**Comparison of global expression changes by *fs(1)h *knockdown by RNAi and BET inhibitor treatment**. (**A**) Western blots indicate efficient FSH protein depletion in S2-DRSC cells after 3 days of dsRNA-treatment. Numbers above lanes mark samples from two biological replicates done in parallel. (**B**) Differential expression analysis by RNA-seq. The upper row shows MA plots comparing gene expression in FSH knockdown cells (DRSC29017 and FSH-L) and cells treated with control dsRNA (eGFP). The lower row shows volcano plots of the same data, illustrating the selection of differentially expressed genes according to a combined *P *value, fold change cut-off. Blue lines indicate the chosen two-fold gene expression cut-off. The red lines illustrates the 5% significance level according to the adjusted *P *value <0.05 computed using DESeq's multiple testing procedure. The number of genes passing both selection criteria are given on top.

Our expression profiling of JQ1-treated *Drosophila *cells showed a fast and widespread down-regulation of transcription. In contrast to mammalian cell culture systems, *Drosophila *offers the opportunity to knockdown BET function by targeting a single gene. We therefore decided to compare the transcriptional response between FSH knockdown and small molecule inhibition. RNAi effectively depleted FSH isoforms from dsRNA-treated S2-DRSC cells within 3 days (Figure 10A). By targeting either both isoforms, or exclusively FSH-L, we intended to partially deconvolute their contribution to transcriptional control. This is of special interest, since the CTM connects FSH-L to the pTEF-b pathways, whereas FSH-S lacks this protein interaction domain [37]. For both knockdowns we monitored gene expression changes using RNA-seq. Subsequently we identified differentially expressed genes by comparing FSH knockdown conditions (two replicates each) with control treatments (eGFP specific dsRNA). For both knockdowns only few genes showed >2-fold differential expression (Figure 10B). In addition, we observed comparable numbers of up- and down-regulated genes. Overall, the selected targeting of FSH-L still showed a weaker effect on transcription, than a complete depletion of both isoforms.

We therefore conclude that FSH knockdown does not induce the drastic transcriptional changes that we observed by small molecule inhibition. We believe that the deviating results are caused by the limited time resolution inherent to RNAi experiments, which in general do not allow measuring the primary response of the gene regulatory network (see Discussion for details).

## Discussion

Experiments using double mutants of the TrxG genes *ash1 *and *fs(1)h *demonstrated a strong genetic interaction, whereas attempts to demonstrate a biochemical interaction remained unsuccessful so far. By purifying tagged ASH1 from a stable cell line, we discovered the missing physical connection between the two transcriptional activators. Indeed, the study by Chang *et al*. [17] attempting to demonstrate this interaction by FSH-S purification might have failed because of inappropriate purification conditions, or deviating starting material with respect to our study.

In line with our interaction proteomics results we show that ASH1 and FSH extensively co-localize on chromatin. Both proteins jointly target active gene promoters irrespective of gene function. Intron-rich, active genes tend to display ASH1and FSH signal distal to the TSS. Contrasting to our results, two recently published ChIP-chip studies have reported that ASH1 forms broad domains encompassing fully activated PcG target genes, or is bound at TSSs maintained in a 'balanced' state [10,11]. This balanced state is distinguished by the co-existence of PcG and TrxG proteins as well as production of full-length mRNA. The investigators deduced from these results that ASH1 cannot be a general transcriptional activator, but instead functions as specific antagonist of PcG-mediated repression. This conclusion was backed up by earlier genetic experiments showing that ASH1 function is dispensable for *Ubx *activation in the absence of PcG-mediated repression [3]. Our ASH1 chromatin maps unequivocally identify ASH1 binding to genes as being highly predictive for transcriptional activity. In addition, we cannot confirm that this behavior is in any sense restricted to a defined set of target genes, such as genes under PcG-control. On the contrary, we detected strong ASH1/FSH signals at housekeeping genes, for instance *Cyp12c1 *(Figure 6C) and *Spf45 *(Figure 8A). We could extend the list by further components of cell metabolism like ribosomal subunits or histone genes (not shown). These findings strongly argue in favor of a general involvement of ASH1 and FSH in transcription. Support for this notion comes from a study examining the chromatin localization of ASH1L in a panel of human cell lines. By applying ChIP-qPCR the authors could show that ASH1L associates with the transcribed region of all active genes examined [33]. In addition, the distribution of ASH1L in transcribed chromatin was found to resemble that of H3K4me3. Our genome-wide, high resolution mapping study in fly cells comes to similar conclusions.

Missing ASH1 signals at active transcription units have been used to argue against a general involvement of ASH1 in transcription. Our analysis of the S2-DRSC chromatin landscape suggests that previous mapping studies failed to detect these signals and therefore jumped to conclusions based on incomplete evidence. A reasonable explanation for the enhanced sensitivity of our mapping study might be the employment of next-generation sequencing technology instead of microarrays. Direct comparison of ChIP-chip and ChIP-seq has shown that ChIP-seq generally produces profiles with better signal-to-noise ratio and allows detection of more and narrower peaks [34]. Still, the lower measurement sensitivity/resolution is not sufficient to explain the magnitude of differences. Our investigation of the antibody Q4177, generated on behalf of the modENCODE consortium, suggests that poor target reactivity of the immunoreagent might be the major explanation for missed ChIP signals in the study by Kharchenko *et al*. [11] (Figure 1D). Schwartz *et al*. [10] however partially relied on the same antibodies as we did.

Regarding the absolute number of reported binding sites we would like to note that we used a peak detection algorithm (MACS) that was developed to map human transcription factor (TF) binding sites in ChIP-seq data (see Materials and methods for details). Sequence specific TFs typically generate sharply restricted, Gaussian-like ChIP-seq signals. Therefore, the algorithm was ideally suited to detect ASH1, FSH signals at TSS. At the same time, it tends to break up more widely distributed signals, like the ones we described for intro-rich active genes, into closely spaced peak clusters. Hence, the absolute number of peaks reported in this study does not reflect the number of functional elements bound by ASH1 and FSH in a one-to-one correspondence.

The second major argument against a general involvement of ASH1 in transcription was provided by the *Ubx *activity in *ash1/E(z) *double-mutant mitotic clones, located in- and outside of the endogenous *Ubx *expression domain [3]. In our opinion, this observation does not directly contradict the co-activator hypothesis favored by our data. It is conceivable that ASH1 and other TrxG proteins only modulate the transcriptional output, rather than being absolutely required for transcription. The incomplete, stochastic loss of *Ubx *activity in embryonic tissues devoid of ASH1 protein already argues against such a strict requirement [3,29], and studies of heat shock response have already demonstrated a transcription modulating function for TRX [35]. The latter finding is especially intriguing, because *trx/E(z) *double-mutant clones show *Ubx *reactivation patterns similar to their *ash1/E(z) *counterparts [3].

Our unsupervised clustering of genes according to their combined TrxG/PcG binding profiles identified a class of transcription units with concurrent high PRC1 and TrxG enrichments at the promoter. In contrast to the 'balanced' state introduced by Schwartz *et al*. [10], these genes do not generate a transcript. We argue that the existence of this 'PcG-repressed' class provides additional evidence against the anti-repressor hypothesis. Instead, we favor the idea that fully installed PcG-repression needs to overcome the general co-activator function of TrxG proteins. We have previously identified Polycomb (Pc) interacting proteins by *in-vivo *biotinylation tagging [36]. Unexpectedly, this approach revealed FSH to interact with Pc in the *Drosophila *embryo. Our current analysis of FSH chromatin occupancy now shows that PRC1 and FSH co-localize at repressed promoters and intergenic gene regulatory elements, although they are known to fulfill antagonistic functions. Mechanistically this might suggest that PRC1 silences genes by blocking the elongation-promoting functions of FSH, for instance via the pTEF-b pathway. In the light of these deliberations it is sensible that PcG-bound promoters do show elevated levels of promoter proximal pausing, as we have reported earlier [28]. Future experiments using defined *in-vitro *transcription systems in conjunction with reconstituted PRC1 and FSH might shed light on the mechanistic details of this interaction.

Our data provides evidence that FSH promotes the transcriptional activity of genes. How might this work at a mechanistic level? The human FSH-L homolog BRD4 proteins contain a conserved pTEF-b interacting domain in the very C-terminus, which is sufficient to directly recruit pTEF-b to target genes [37]. Following recruitment, pTEF-b triggers the transition from early elongation (promoter-proximal pausing) to productive elongation by phosphorylating Serine 2 residues in the RNA polymerase II CTD [38]. In addition, pTEF-b has been shown to phosphorylate DISF and NELF which releases the repressive factor NELF and converts DISF into a positive elongation factor. Studies in *Drosophila *have shown that pausing represents a widespread gene regulatory strategy. Our data are in line with this possible mechanism, since displacement of FSH leads to a widespread down-regulation of transcription. A recent study has shown that BRD4 is also able to directly phosphorylate Serine 2 of the Pol II CTD [39]. A kinase activity has also been demonstrated for FSH-S [17]. Using a defined *in-vitro *transcription system, it has been shown that the human BRD2, BRD3 render nucleosomes marked by acetylation permissive to the passage of elongating RNA polymerase II, thereby bypassing FACT requirement [40]. Taken together, FSH might therefore support the passage of two central checkpoints of the transcription cycle: first, it might be critical to release RNA polymerase II into productive elongation by initiating CTD phosphorylation and pausing factor release. Second, it potentially removes nucleosomal barriers in front of the elongating polymerase. In addition, the loss of ASH1 from promoters after FSH removal might indicate that the ASH1-FSH interplay stabilizes the association of ASH1 with chromatin. Additional evidence for cooperation between ASH1 and FSH during gene activation is provided by our recent study, applying regression models in order to predict gene expression based on chromatin binding profiles. In this quantitative modeling framework, the two proteins form an interaction pair [31].

Our JQ1 treatment of *Drosophila *tissue culture cells provides evidence, that transcriptome homeostasis is heavily affected by small molecule inhibition of BET proteins. This observation corresponds to our FSH chromatin map, since we observed a high correlation between FSH localization and gene activity. Exactly the same results have recently been described with respect to BRD4 in human CD4+ T cells and ES cells. ChIP-seq revealed that BRD4 localizes to active promoters and enhancers, and that expression levels are strongly correlated with BRD signal intensity [41]. BRD4 profiles across active human promoters display that BRD4 binds at the TSS. Here we show the same for FSH in *Drosophila *cells. The degree of correlation between BRD4 localization and transcriptional activity even encouraged the authors to predict that 'BRD4 could potentially be used as a genome-wide hallmark of active or poised genes'. Our gene clustering according to TrxG/PcG promoter binding supports this idea. Interestingly, our clustering into active and PcG-repressed genes parallels the human classification into active and poised genes in a sense that poised genes are occupied by BET proteins, but do not produce mRNA. Disruption of BRD4 chromatin binding by JQ1 in CD4+ T cells resulted in reduced expression of more than a thousand genes in CD4+ T cells [41]. Again, we have reporter similar observation using JQ1 treatment in fly cells. In contrast, knockdown of FSH did not result in comparable transcriptional repression. We argue that the deviating results are due to the different natures of the treatment. Small molecule inhibition happens almost instantaneously. By monitoring the system in a time-resolved manner it is possible to untangle primary and secondary effects. Effective depletion of target proteins by RNAi can only be achieved within time scales of days. Therefore RNAi experiments rather disclose a novel steady-stead of the reduced network than primary effects. Similar thoughts should be considered when interpreting expression profiles obtained from zygotic mutants, since maternal gene products are present during development. Like in RNAi-mediated knockdowns it becomes impossible to define a time point that corresponds to functional null situation, but is not governed by secondary effects.

A recent study in erythroid cells shows, that BRD3 is recruited to target promoters by reading an acetyl mark on the transcription factor GATA1, instead of being primarily dependent on histone acetylation [42]. The concentration of FSH signals around TSSs fits the idea that acetylated transcription factors might also contribute to BET protein recruitment in flies.

## Conclusions

There is substantial evidence that TrxG proteins play global roles in transcription. In the future it will be interesting to find out, why this group of proteins genetically scores as being important for the maintenance of the active state of HOX genes, or in other words, what discriminates them from other factors in functional proximity to RNA polymerase. More mechanistic insight how TrxG complexes are connected to transcription machinery will be necessary to answer this question. It might indeed turn out that TrxG complexes 'are among those that have emerged as being important for maintenance for a relatively uninteresting reason e.g., because even relatively subtle changes in the expression of *Drosophila *HOX genes cause homeotic transformations' [1].

## Materials and methods

### Generation of ASH1 expression construct and stable cell line

The *ash1 *coding sequence was PCR-amplified from S2 cell derived cDNA in four overlapping parts (A-D) using the primers listed in Additional file 2, Table 1. Subsequently, each PCR product was inserted into the pENTR/D-TOPO vector for the Gateway Cloning System as outlined in the manufacturer's instructions (pENTR/D-TOPO cloning kit, Invitrogen). The full-length *ash1 *coding sequence was reassembled from these primary clones by three directional subcloning steps, utilizing the restriction sites indicated in Figure 1A. Sanger sequencing confirmed that the derived full-length cDNA matches the ASH1 SwissProt entry [Swiss-Prot:Q9VW15] with the exception of codons T1716 and L1717, which seems to be absent from *ash1 *mRNA in S2 cells (Figure 1C).

In order to generate the ASH1 expression construct outlined in Figure 2A, the Hygromycin resistance cassette (*AccI/SapI *fragment taken from pCoHygro plasmid, Invitrogen) was inserted into the *AccI/SapI*-cut pMT/V5-His A vector backbone (Invitrogen). In addition, the sequence between *EcoRV/HpaI *encompassing MCS to SV40 polyA was replaced by the *EcoRV/PmeI*-excised fragment from pAFW (Drosophila Gateway Collection, available through DGRC). In a subcloning step, this fragment was previously modified by inserting the 8xHis-TEV coding sequence (generated by hybridizing the oligo nucleotides listed in Additional file 1, Table 4 into the *AgeI *site between 3xFLAG and gateway cassette. Finally, the *ash1 *coding sequence was transferred to the inducible pMT/FHW HygRes expression vector by standard gateway cloning according to manufacturer's instructions (Invitrogen).

In order to generate the stable cell line, S2-DRSC cells grown in Schneider's medium incl. 10% FCS were lipotransfected using pMT/FH-ASH1 HygRes according to standard Effectene protocols (Quiagen). Two days post-transfection transformants were selected by Hygromycin B addition to the growth medium (500 µg mL^−1^) and passaged for 4 weeks in the presents of the selecting agent.

### FH-ASH1 purification and identification of co-purified proteins

Expression of tandem tagged ASH1 was induced by adding 500 µM copper(II) sulfate to the growth medium for approximately 16 h. Afterwards cells were scraped in ice cold PBS, pelleted by low speed centrifugation (500 × g) and suspended in N buffer (15 mM HEPES pH 7.4, 10% Sucrose, 0.5 mM EGTA, 60 mM KCl, 15 mM NaCl, Complete Protease Inhibitors EDTA-free). Resuspended cells were lysed by adding Triton X-100 to 0.5% (vol./vol.) for 10 min. Next, nuclei were separated from cytoplasm by passing the lysate through a sucrose cushion (20% sucrose in N buffer) and washing nuclei twice in N buffer. In order to extract nuclear proteins, washed nuclei were transferred to lysis buffer (15 mM TRIS pH 7.4, 300 mM NaCl, 1 mM MgCl, 0.1 mM EDTA, 20 mM Imidazole, 0.1% NP-40, 10% Glycerol, Complete Protease Inhibitors EDTA-free) and sonicated in the Bioruptor (Diagenode) for five to 10 cycles (20 s ON/40 s OFF, high power setting). Afterwards nuclear extract was treated with 150 u mL^-1 ^Benzonase (Novagen) for 10 min and cleared by ultra-centrifugation (100,000 × g for 1 h).

In order to capture 8xHis tagged ASH1 protein, nuclear extract was diluted in IMAC buffer A (50 mM TRIS pH 7.4, 20 mM Imidazole, 100 mM NaCl, 10% Glycerol, Complete Protease Inhibitors EDTA free), adjusted to 300 mM NaCl, and loaded onto a Ni-Sepharose column (HisPrep FF 16/10, GE Healthcare) connected to a FPLC system (AKTApurifier, GE Healthcare). Afterwards non-specifically bound proteins were eliminated by washing the column with 4% IMAC buffer B (50 mM TRIS pH 7.4, 500 mM Imidazole, 100 mM NaCl, 10% Glycerol, Complete Protease Inhibitors EDTA-free) and bound proteins were eluted at 60% buffer B. Eluate fractions were probed for FLAG-tagged ASH1 by western blot, pooled accordingly, and adjusted to 100 mM NaCl by diluting pooled material in IEX buffer A (50 mM TRIS pH 7.4, 10% Glycerol, Complete Protease Inhibitors EDTA-free). Next, material was loaded onto an anion exchange column (SOURCE 15Q 4.6/100 PE, GE Healthcare) and washed using 10% IEX buffer B (50 mM TRIS pH 7.4, 1 M NaCl, 10% glycerol, Complete Protease Inhibitors EDTA-free). Bound material was eluted by a linear gradient from 10 to 100% IEX buffer B over 10 column volumes again monitoring FLAG-tagged ASH1 by western blot. ASH1-containing eluate fractions were pooled, adjusted to 0.05% NP-40 and incubated overnight with 100 µL FLAGM2 agarose (Sigma). Next, agarose was washed several times using TBS, 0.05% NP-40 and transferred to Mobicol spin columns for eluting bound material by 200 µg mL^-1 ^3xFLAG peptide (Sigma) dissolved in wash buffer. Eluted proteins were precipitated in 20% TCA (w./vol.) by high-speed centrifugation, washed using 10% TCA and ice-cold Acetone, and air-dried. Next, dried proteins were resuspended in reduction buffer (500 mM TRIS pH 8.6, 6 mM Guanidin-HCl), reduced and alkylated by subsequent addition of TCEP (100 mM) and Iodoacetamid (250 mM), respectively. Then proteins were digested in digestion buffer (50 mM TRIS pH 7.4, 5 mM CaCl_2_, 2% ACN) by addition of 0.1 mg mL^-1 ^Trypsin Gold (Promega). Tryptic peptides were separated using online reverse-phase chromatography and electrosprayed into a LTQ Orbitrap Velos tandem mass spectrometer (Thermo Scientific). MS spectra were recorded at 60,000 R and most intense peptide ions were selected for CID fragmentation and recording of product ion spectra. MS/MS spectra were matched against UniProtKB release 2011.08 using the Mascot search engine (Matrix Science), assuming the digestion enzyme Trypsin and up to one missed cleavage. Mascot was searched with a fragment ion mass tolerance of 0.5 Da and parent ion tolerance of 5 ppm. Iodoacetamide derivative of cysteine was specified in Mascot as a fixed modification. S-carbamoylmethylcysteine cyclization (N-terminus) of the n-terminus, deamidation of asparagine and glutamine and oxidation of methionine were specified in Mascot as variable modifications. Scaffold 3.3.1 (Proteome Software) was used to validate MS/MS based peptide and protein identifications. Peptide identifications were accepted if they could be established at >95.0% probability as specified by the Peptide Prophet algorithm [43]. Protein identifications were accepted if they could be established at >99.0% probability and contained at least three identified peptides. Protein probabilities were assigned by the Protein Prophet algorithm [44]. For complete results see Additional file 1.

### Generation of FSH expression constructs and co-immunoprecipitation

The FSH-S coding sequence was PCR-amplified from the Drosophila Gold Collection clone LD26482 (available through DGRC) using primers listed in Additional file 2, Table 1. The PCR-product was afterwards inserted into pENTR/D-TOPO as outlined in the manufacturer's instructions (pENTR/D-TOPO cloning kit, Invitrogen). In order to generate the HA-tagged expression construct the FSH-S ORF was transferred to pAHW (Drosophila Gateway Collection, available through DGRC) using standard gateway cloning. In addition, the ASH1 fragments A-D were transferred to the 3xFLAG-tagged pAFW plasmid (Drosophila Gateway Collection). For co-immunoprecipitation S2-DRSC cells were co-transfected with pair-wise combinations of pAH-FSH-S and pAF-ASH1 A-D by using standard Effectene (Qiagen) lipotransfection protocols. Two days post-transfection whole cell lysates were prepared using RIPA lysis buffer and tagged ASH1 fragments were immunoprecipitated by FLAGM2 antibody (Sigma) coupled to Protein G Dynabeads (Invitrogen). Precipitated proteins were eluted by boiling Dynabeads at 70°C in 1xLDS sample buffer (Invitrogen) for 10 min. Immunoblotting of eluted proteins and nuclear extracts was conducted using Novex TA 3-8% gradient gels and iBlot transfer system according to manufacturer's instructions (Invitrogen). For immunodetection of HA-tagged FSH-S HA.11 antibody (Covance) was used at 1:2,000 in PBS, 0.1% Tween-20, 5% dry milk powder followed by anti-mouse IgG-HRP (GE Healthcare) at 1:10,000 in PBS, 0.1% Tween-20, 5% dry milk powder. ECL detection of proteins was conducted using ECL substrate and X-ray films from GE Healthcare.

### FSH knockdown by RNAi

PCR products serving as templates for *in-vitro *transcription were generated from pENTR/D-TOPO FSH-S using T7 recognition sequence flanked primers listed in Additional file 2, Table 3. PCR amplicons DRSC18778, DRSC29017 were taken from the DRSC reagent database [45]. The FSH-L specific amplicon was designed using E-RNAi [46]. Purified PCR products were transcribed to dsRNA and cleaned using the MEGAscript RNAi Kit according to manufacturer's instructions (Ambion). RNAi was carried out according to standard bathing protocols in 6-well plate format (Drosophila RNAi Screening Center, Harvard Medical School) by adding 15 µg of dsRNA to each well before platting S2-DRSC cells in serum free Schneider's medium and starving cells for 30 min.

### FSH antibody preparation

Serum from rabbits immunogenized with FSH-specific antigens was kindly provided by Igor Dawid (National Institute of Child Health and Human Development). The antigen used for rabbit ID166 corresponds to the XbaI/PstI fragment from clone e1.20 and the antigen injected into rabbit ID173 is derived from the EcoRI/HindIII fragment of clone e4.1, both described in [16]. Generation of β-Gal/FSH-S fusion constructs, protein expression, and immunization was done in the laboratory of I. Dawid as described in [17]. Immunoglobulin G purification from rabbit serum using Protein A Sepharose CL-4B (GE Healthcare) was carried out as described in [47], Basic Protocol 2. In order to validate the antibody specificity, we performed immunoblotting of S2-DRSC lysates depleted of FSH protein by RNAi as described before (Figure 4C). In order to reduce high background signals, FSH-specific antibodies were diluted in high-salt PBS (300 mM NaCl), 1% Tween-20, 5% dry milk powder.

### Quantification of LPS-inducible gene expression under JQ1 treatment

One hour before lipopolysaccharide (LPS) was added to the growth medium S2-DRSC cells were pretreated using either JQ1 or DMSO as described before. After LPS addition (5 µg mL^-1^) cells were lysed at indicated time points using Trizol (Invitrogen). In order to quantify the transcriptional response of known LPS-inducible genes (*CecA1*, *CecB*) total RNA was extracted, reverse transcribed (First Strand cDNA Synthesis kit, Fermentas), and TURBO DNase (Ambion) treated as outlined in the manufacturer's instructions. Quantification of cDNA was done by qPRC using SYBR Green chemistry and primers listed in Additional file 2, Table 2 on the LightCycler 480 thermocycler (Roche Diagnostics). For calculating target gene levels relative to the house keeping gene *ATPsyn-Cf6 *the ΔΔct method including efficiency correction was used. Determination of c_t _values was done by calculating the second derivative maximum of the SYBR green signal.

### Histone peptide pull-down assays

The V5-tagged FSH-S expression construct was created by transferring the FSH-S ORF from pENTR/D-TOPO to pcDNA6.2/V5-DEST using gateway cloning according to manufacturer's instructions (Invitrogen). Nuclear extract from HEK293 cells lipotransfected with pcDNA6.2/V5-FSH-S was prepared 2 days post-transfection using Active motif's Nuclear Extract Kit as outlined in the corresponding manual. For histone peptide pull-down assays 10 µL of nuclear extract (equivalent to 2 × 10^6 ^cells) was diluted in binding buffer (50 mM TRIS pH 8.0, 150 mM NaCl, 5 mM MgCl, 0.1% NP-40, Complete Protease Inhibitors EDTA-free) and incubated with 20 µg Acetyl-Histone H4 (Lys5, 8, 12, 16) peptides (Millipore, Catalog # 12-379) in the presence or absence of 1 µM JQ1. Afterwards biotinylated peptides were precipitated using Streptavidin Dynabeads M-270 (Invitrogen) and washed several times in binding buffer. Peptide-bound protein was eluted by boiling Dynabeads in 1xLDS sample buffer (Invitrogen) and immunoblotted together with nuclear extracts as described for coIP assays. V5-tagged protein was detected using anti-V5 antibody (Invitrogen) at 1:5,000 in PBS, 0.1% Tween-20, 5% dry milk powder, followed by anti-mouse IgG-HRP (GE Healthcare) at 1:10,000 in PBS, 0.1% Tween-20, 5% dry milk powder, ECL substrate, and Hyperfilm (GE Healthcare).

The BD1-BD2 coding sequence was PCR amplified from the vector LD26482 and cloned into pNIC28-Bsa4 (Structural Genomics Consortium) applying ligation independent cloning (LIC). The resulting plasmid was used to express 6xHis-tagged BD1-BD2 in BL21(DE3) E. coli. Cell lysate was prepared by sonicating cells in 20 mM Tris-HCl pH 8.0, 1 mM PMSF, 1mg/ml Lysozyme, 0.05% Triton X-100 for 15 min (30 s ON/30 s OFF) in a water bath sonicator followed by high speed centrifugation. BD1-BD2 was purified from cleared lysate by IMAC on Ni-NTA Superflow (Qiagen) deploying an imidazole step-gradient (50, 100, 150, 250 mM). BD1-BD2 containing eluate fractions were monitored by Coomassie-stained SDS-PAGE and concentrated in 25 mM HEPES pH 8.0, 150 mM Nacl using 10 NMWL centrifugal filter devices (Millipore). For BD1-BD2 pull-downs approximately 3 µg of purified protein was incubated with 35-40 µM of histone tail peptide in 25 mM HEPES pH 8.0, 150 mM NaCl in the presence or absence of 10 µM JQ1. After 1 h of incubation at 4°C biotinylated peptides were captured on streptavidin magnetic beads and washed three times in binding buffer. Precipitated proteins were eluted by boiling magnetic beads in 1x LDS buffer. Eluted proteins and input samples were immunoblotted using anti His-probe (H-3) HRP (Santa Cruz Biotechnology) and standard ECL reagents.

### Analysis of protein binding to chromatin by ChIP-seq

Chromatin immunoprecipitation followed by next-generation sequencing (ChIP-seq) was essentially done as described in [28] using formaldehyde cross-linked chromatin from 2.5 × 10^7 ^S2-DRSC cells per ChIP. In order to assure data comparability across ChIP-seq experiments, all chromatin samples were taken from the same chromatin batch. This chromatin batch is identical to the one we used previously to generate ChIP-seq data for PC, PH, PSC, and TRX-C.

ASH1-bound chromatin was enriched using two antibody preparations provided by the lab of F. Sauer (University of California, Riverside). Anti-ASH1-N recognizes an antigen located in the amino-terminus of ASH1, while the anti-ASH1-C specific antigen resides in the carboxy-terminal portion of the protein. Both polyclonal antibodies have been raised in rabbits. Since different dsRNA treatments of S2-DRSC cells did not induce detectable ASH1 knockdown (data not shown), we investigated antibody specificity by expressing ASH1-GFP fusion proteins in S2-DRSC cells and immunoblotting of cell lysates (Figure 1B). FSH-bound chromatin was enriched by the anti-FSH and anti-FSH-L antibody preparations described above.

Sequencing libraries were prepared from 10 ng of immunoprecipitated DNA using the ChIP-seq DNA Sample Prep Kit (Illumina), including size selection of preamplified fragments on agarose gels (200 bp +/- 30 bp). For quality control, the size distribution of the final libraries was assayed on the Agilent BioAnalyzer 2100 using High Sensitivity DNA microfluidic chips. Each library was sequenced for 36 cycles in a single-end run on the Genome Analyzer IIx (Illumina). Sequencing yielded approximately 20 × 10^6 ^quality filtered reads per library (Illumina Chastity Filter), corresponding to a mean genome coverage of approximately 5x. Short reads (36 bp) were aligned to the *Drosophila *reference genome (BDGP Release 5) using Bowtie 0.12.7 and the following parameters: -n 2, -m 20, -k 1, --best [48]. Since anti-ASH1-N and anti-ASH1-C target the same protein isoform, we decided to merge the corresponding alignments prior to downstream analysis. The same was done for the FSH-specific alignments, since we could not detect mayor differences between these datasets (data not shown). Merging and indexing of alignments was done using SAMtools 0.1.9 [49]. Regions showing significantly enriched read coverage compared to input chromatin (also referred to as 'peaks') were calculated using MACS 1.4.0 and the following parameters: band width, 300; model fold, 10 to 30; *P *value cut-off, 1x10^-5 ^[50]. Read coverage profiles were calculated from aligned reads by shifting and extending reads according to the fitted MACS models. The distance calculation between peak intervals and TSS was carried out using the R/Bioconductor package ChIPpeakAnno (Release 2.9) and basic R functions [51]. Interval based co-localization analysis was done by comparing MACS peak lists with BEDtools 2.10.0 [52]. The compilation of high-confidence PRC1 sites was constructed by intersecting peak lists for PC, PH, and PSC using BEDtools. PC, PH, and PSC specific peak list were compiled using MACS on our previously published PcG ChIP-seq dataset available through NCBI's Gene Expression Omnibus [53]. Metagene profiles were created using the R/Bioconductor package GenomicRanges (Release 2.9). In short, based on alignments, coverage vectors for each chromosome were calculated. From these vectors, all subvectors were extracted that correspond to ORFs of known protein coding genes having well separated TSSs (min. distance to next TSS = 1 kb). Next, kernel density functions were calculated from these subvectors and sampled at 500 equally spaced points in order to create read density estimates at positions relative to ORFs. Finally, the position-wise mean densities were calculated after grouping genes according to expression level and plotted along relative positions in order to obtain metagene profiles. In order to create promoter profiles, coverage vectors spanning all non-overlapping 1-kb windows centered at known TSS were calculated. Position-wise mean read coverage was calculated and plotted along the relative position to obtain coverage profiles. Quality-filtered reads, MACS peak lists, and coverage profiles have been deposited in NCBI's Gene Expression Omnibus and are accessible through GEO Super Series accession number GSE36450 [54].

### Gene expression analysis by RNA-seq

For JQ1 treatment of exponentially growing S2-DRSC cells lyophilized JQ1 was dissolved in DMSO (10 mM) and added to the culture medium at a concentration of 10 µM. RNA extraction from S2-DRSC cells was done using Trizol reagent according to manufacturer's instruction for adherent cells (Invitrogen). Barcoded sequencing libraries were prepared from 4 µg of total RNA according to Illumina's TruSeq protocol including polyA-enrichment, mixed in equal proportions and sequenced together on a single HiSeq2000 lane (Illumina) using a 50-cycle single-end run.

Alignments of RNA-seq reads were generated using the splice-junction-aware aligner TopHat [55] and *Drosophila *gene models originating from Ensembl release 64 (default parameters). All reads uniquely mapping to gene models were counted by the HTSeq-count script (HTSeq developed by Simon Anders, EMBL Heidelberg) and the count statistic was forwarded to bioconductor package DESeq [56]. Since the standard size factor estimation of DESeq produced inappropriately adjusted counts, an alternative normalization procedure was applied, assigning the 100 most abundant genes in the control condition as normalization index. The principle behind this strategy is comparable to qPCR normalization using a house keeping gene index.

Demultiplexed RNA-seq reads and raw gene counts have been deposited in NCBI's Gene Expression Omnibus and are accessible through GEO Super Series accession number GSE36450 [57].

### Computation of ChIP-seq enrichments at promoters

ChIP-seq and input reads were counted on 1kb windows +/- 500 bp around *N *= 13,254 unique TSS defined in the ensGenes table of the UCSC browser with the pysam python module. Subsequently, the relative frequencies *p_i _*= *X_i_ /m *of ChIP counts *X_i _*on TSS *i *were normalized by a rescaling of the library size *m *to maximize the number of TSS where the relative ChIP frequency was identical to that of the corresponding value *q_i _*= *Y_i_ /n *in the input experiment. The symbol *Y_i _*denotes the read count in interval *i *in the input experiment and *n *the input library size. For each TSS and ChIP experiment, a binomial likelihood ratio test was computed testing the null hypothesis *p_i _= q_i _versus *the alternative *p_i _≠ q_i_*, and the sum over all TSS of the resulting *P *values was maximized numerically over *m*. Normalized enrichments were then computed as *p_i_ /q_i_*. Data normalization and enrichments were computed in R.

### Gene clustering according to TrxG/PcG ChIP-seq profiles at promoters

The sparse Gaussian mixture model (sGMM) is a probabilistic mixture model of multivariate Gaussian distributions, in which the covariance matrices are estimated under an *L*_1_-penalty favoring sparse solutions [59]. The model was fitted to the set of logarithmic TrxG/PcG ChIP-seq enrichments at known transcription start sites defined above.

Let *x^i ^∈ R^n ^*be a vector of ChIP-seq enrichments from different antibodies in TSS window *i *and *ϕ(x^i^; µ, Σ) *denote the density of a multivariate Gaussian with mean *μ *and covariance Σ. An sGMM with *m *mixture components (classes) is defined by the likelihood function

(1) $$\begin{array}{ccc} L\left(x^{i};{\left\{\pi_{k},\mu_{k},\underset{k}{\sum }\right\}}_{k=1}^{m}\right) & = & \overset{m}{\underset{k=1}{\sum }}\pi_{k}\phi \left(x^{i};\mu_{k},\underset{k}{\sum }\right)\text{exp}\left(-\rho ∥\overset{-1}{\underset{k}{\sum }}∥1\right)\\ & = & \overset{m}{\underset{k=1}{\sum }}\pi_{k}{\tilde{\phi }}_{k}\left(x^{i}\right) \end{array}$$

where ρ ≥ 0 defines the penalization strength, ||Σ*_k_^-1^*||_1 _is the *L*_1_-norm of the inverse covariance matrix, and ${\tilde{\phi }}_{k}\left(x^{i}\right)=\phi \left(x^{i};\mu_{k},\Sigma_{k}\right)\text{exp}\left(-\rho ∥\Sigma_{k}^{-1}{∥}_{1}\right)$.

The model parameters *π_k_*, *μ_k_*, and Σ*_k _*were estimated iteratively by the expectation maximization (EM) algorithm, where in each step the inverse covariance matrices Σ*_k_^-1 ^*are computed from the weighted empirical covariances $S_{k}=\Sigma_{i=1}^{N}\gamma_{k}^{i}{\left(x^{i}-\mu_{k}\right)}^{\text{T}}\left(x^{i}-\mu_{k}\right)$ with the graphical LASSO algorithm [60,61]. Here $\gamma_{k}^{i}=\pi_{k}{\tilde{\phi }}_{k}\left(x^{i}\right)/\Sigma_{t}\pi_{t}{\tilde{\phi }}_{t}\left(x^{i}\right)$ denote the posterior probabilities of observation *x^i ^*belonging to class *k*, and *N *is the number of TSS. For *ρ *= 0, the sparse GMM is equivalent to a regular Gaussian mixture model with full covariance matrices, while for large values of *ρ *one enforces a GMM with diagonal covariance matrices, similar to the k-means algorithm. Clustering of the TSS is achieved by the Bayes estimate assigning *x^i ^*to the class *k *that maximizes *γ_k_^i^*. This procedure also allows for predicting the classes of new TSS by computing their class probabilities according to Eq. (1).

A three-component sGMM was fitted to normalized ChIP enrichment values as stated above. The unpenalized sGMM had a tendency to overfit the third component into the shoulder of the cluster of highly enriched Ash1/Fsh TSS, instead of separating highly enriched PRC1 TSS. Under a penalization of *ρ *= 0.5, the model yielded three distinct classes with diagonal covariance matrices, except for a cluster of correlated high PRC1 enrichments. These classes overlapped 94% with the results of a k-means clustering, confirming the robustness of the sGMM estimation. TSS clusterings were computed in R.

## Abbreviations

aa: amino acid; AMP: antimicrobial peptide; BET: bromodomain and extraterminal proteins; BX-C: bithorax complex; cDNA: complementary DNA; ChIP-chip: chromatin immunoprecipitation followed by microarray analysis; ChIP-qPCR: chromatin immunoprecipitation followed by quantitative polymerase chain reaction; ChIP-seq: chromatin immunoprecipitation followed by next-generation sequencing analysis; CTM: C-terminal motive; DGRC: Drosophila Genomics Resource Center; DRSC: Drosophila RNAi Screening Center, Havard Medical School; ES: embryonic stem; FH-ASH1: 3xFLAG, 8xHis-tagged ASH1 protein; FLAG-AC: FLAG affinity chromatography; HMT: histone methyltransferase; IEX: ion exchange chromatography; IMAC: immobilized metal affinity chromatography; LPS: lipopolysaccharide; MS/MS: tandem mass spectrometry; MW: molecular weight; ncRNA: non-coding RNA; ORF: open reading frame; PcG: Polycomb group; Pol II CTD: RNA Polymerase II C-terminal domain; PRC: Polycomb repressive complex; qPCR: quantitative polymerase chain reaction; RNAi: RNA interference; RNA-seq: next-generation sequencing of RNA; sGMM: sparse Gaussian mixture model; TSS: transcription start site; TrxG: Trithorax group.

## Authors' contributions

TK carried out all experiments if not stated otherwise, performed computational analysis of ChIP-seq and RNA-seq data, participated in statistical data analysis, and drafted the manuscript. MG performed statistical data analysis with assistance by NB and participated in manuscript preparation. TS performed phylogenetic analysis and ZH contributed coIP experiments. DH performed MS/MS measurements and data analysis. CB conceived the study and helped TK to acquire and interpret data. RP helped to interpret data and revised the manuscript. All authors read and approved the final manuscript.

## Supplementary Material

Additional file 1 **ESI-MS/MS analysis overview**. Spreadsheet contains analysis results of ESI-MS/MS experiments. Click here for file

Additional file 2 **DNA oligonucleotides used in this study**. Tables listing oligos used for PCR, molecular cloning, and qPCR. Click here for file
