# Supplementary material for: The BET protein FSH functionally interacts with ASH1 to orchestrate global gene activity in Drosophila
Source: Genome Biol. 2013 Feb 25;14(2):R18. doi: 10.1186/gb-2013-14-2-r18 (PMC4053998; doi:10.1186/gb-2013-14-2-r18)
Supplement: Additional file 2 — DNA oligonucleotides used in this study. Tables listing oligos used for PCR, molecular cloning, and qPCR. [file gb-2013-14-2-r18-S2.PDF]

Additional file 2, Kockmann et al. 2012

Table 1: PCR primers used for cloning of ASH1 and FSH-S ORFs. All forward primers contain the 5-prime cacc sequence (lower case) for directional gateway cloning.

name	sequence	mate
ASH1Afor	cac cAT GAG CTG TAG CCA AAA TGA G	ASH1Arev
ASH1Arev	GGA CGC TCT CCT GGA GTC AAA AG	ASH1Afor
ASH1Bfor	cac cGA TGA TCA CAA GTT GCC G	ASH1Brev
ASH1Brev	AAC TGG CAT AGT CCT GCG GAC C	ASH1Bfor
ASH1Cfor	cac cAT TCC TAC CGA GCA CGA TC	ASH1Crev
ASH1Crev	TCC TCC TCT GAC GAA AGC ACT TCC	ASH1Cfor
ASH1Dfor	cac cTC GTT GCA GAG CTT TAA ACC	ASH1Drev
ASH1Drev	TTA TGT TGA GTT GGC CGT AGA ACT GC	ASH1Dfor
FSH-Sfor	cac cAT GTC GTC CAG TGA GCC ACC	FSH-Srev
FSH-Srev	CTA ACC TGC TTC ACT GTC GCT CG	FSH-Sfor

Table 2: qPCR primer used for measuring expression of AMP transcripts.

name	sequence	mate
CecA1for	GAC CTC ACT GCA ATA TCA ATA TCT TT	CecA1rev
CecA1rev	GGT GAT GGC CAG AAT GAG AG	CecA1for
CecBfor	AAT CCA GGT CCT CGG AAT C	CecBrev
CecB1rev	AAT AAG AGA AAT GAG CGG GTC	CecBfor
ATPsyn-Cf6for	AGA AGA GCG CCG GTG GCA A	ATPsyn-Cf6rev
ATPsyn-Cf6rev	AAC ATC GGG GAA CTG GAA TTC G	ATPsyn-Cf6for

Table 3: RNAi primers used for the generation of T7-flanked PCR products.

name	sequence	mate
T7-DRSC18788S	TAA TAC GAC TCA CTA TAG GGT TGA TGT CGA GCG ACA AC	T7-DRSC18788rev
T7-DRSC18788R	TAA TAC GAC TCA CTA TAG GGG CCG GTG CTG GCT TT	T7-DRSC18788for
T7-DRSC29017S	TAA TAC GAC TCA CTA TAG GTA ATC GGT GGT ATG GAC GGT	T7-DRSC29017rev
T7-DRSC29017R	TAA TAC GAC TCA CTA TAG GAA GCG GCT GGA GAA CAA CTA	T7-DRSC29017for
T7-FSH-Lfor	TAA TAC GAC TCA CTA TAG GGG GCA GCA GCA CAA CAA GAA T	T7-FSH-Lrev
T7-FSH-Lrev	TAA TAC GAC TCA CTA TAG GGG TAG AAC CGG CGC TGA ACT	T7-FSH-Lfor
T7-eGFPfor	TAA TAC GAC TCA CTA TAG GAC CCT CGT GAC CAC CCT GAC CTA C	T7-eGFPrev
T7-eGFPrev	TAA TAC GAC TCA CTA TAG GGG ACC ATG TGA TCG CGC TTC TCG T	T7-eGFPfor

Table 4: Oligonucleotides used for the integration of 8xHis-TEV sequence into FLAG- tagged gateway cassette.

name	sequence	mate
AgeI-8xHis-TEVfor	CCG GCA TCA CCA TCA CCA TCA CCA TCA CGA AAA CCT GTA TTT TCA GGG	AgeI-8xHis-TEVrev
AgeI-8xHis-TEVrev	CCG GCC CTG AAA ATA CAG GTT TTC GTG ATG GTG ATG GTG ATG GTG ATG	AgeI-8xHis-TEVfor
